# Ascending and descending motor pathways converge in the centrolateral nucleus of the thalamus

**DOI:** 10.21203/rs.3.rs-8264428/v1

**Published:** 2026-01-19

**Authors:** N Naeem, SP Masterson, AS Slusarczyk, ME Bickford

**Affiliations:** Anatomical Sciences and Neurobiology, University of Louisville, Louisville KY, 40292

## Abstract

Corollary discharge (CD) signals are copies of motor commands that inform the brain of impending movements. Dysfunction in CD has been implicated in a variety of disorders, from schizophrenia to Parkinson’s disease. In the current study, we used intersectional viral tracing techniques, electron microscopy, and dual opsin optogenetics to investigate potential CD circuits in mice. We focused on the centrolateral (CL) nucleus of the thalamus to relate our findings to previous studies of CD carried out in primates. We found that single neurons in the CL integrate ascending inputs from premotor neurons in the superior colliculus with descending inputs from neurons in layer 5 of the motor cortex; these integrated signals are subsequently sent to the striatum and motor cortex. These results suggest that CL circuits may detect the relative timing of cortical and subcortical movement commands, providing an important feedback loop for ongoing adjustments of movement planning and initiation.

## Introduction

The superior colliculus (SC) is an evolutionary conserved part of the midbrain that controls sensory-driven orienting and defensive behaviors (May 2006). The SC is a laminated structure; the superficial (dorsal) layers receive input from visual areas while the deep (ventral) layers receive auditory, somatosensory, and motor inputs and contain premotor output neurons (Basso et al., 2021). Two major descending premotor pathways arise from the deep layers of the SC: an ipsilateral pathway is involved in defense and escape behaviors (Dean et al., 1989; Redgrave et al., 1993), while a contralateral pathway crosses the midline in the predorsal bundle (PDB) to innervate brainstem gaze centers and the spinal cord to initiate orienting head and body movements (Isa et al., 2020; Wheatcroft et al., 2022; Bickford and Hall 1992). The axons of PDB cells branch to innervate the ipsilateral thalamus (Bickford and Hall 1989; Masullo et al., 2019) suggesting that they send movement commands both upstream and downstream.

A copy of a movement command is known as “corollary discharge” (CD) or “efference copy”. Such signals are critical for distinguishing external from self-generated sensory signals (Crapse et al., 2008; Fukutomi and Carlson 2020). In primates, inactivation of the thalamic mediodorsal (MD) nucleus impairs the ability of animals to correctly execute consecutive saccades (Sommer and Wurtz 2002). This deficit is thought to occur because receptive fields in cortical targets of the MD fail to correctly update to reflect the location of anticipated eye movements. Following MD inactivation, a deficit in receptive field updating was detected within the frontal eye fields (FEF), a cortical area involved in generating saccadic eye movements (Sommer and Wurtz 2006). The FEFs project to the stratum griseum intermediale (SGI) of the SC where PDB cells reside, as well as brainstem gaze centers, and thalamic nuclei that receive input from the SGI (Stanton, Goldberg, and Bruce 1988). Therefore, cortical and subcortical regions presumably collaborate to plan, initiate, and modify gaze shifts, but how these pathways interact at the single-cell level has not previously been explored.

In the current study, we took advantage of a mouse line that expresses Cre-recombinase in a subset of premotor neurons in the SGI, recently described by Masullo et al., 2019. These glutamatergic neurons express *paired-like homeodomain transcription factor-2* (Pitx2^ON^) and account for almost half of the glutamatergic neurons in the SGI. The axonal projections of Pitx2^ON^ cells descend to cross the midline in the PDB and innervate brainstem gaze centers; they also ascend to innervate thalamic targets. Moreover, *in*-*vivo* optogenetic activation of Pitx2^ON^ cells initiates precise head movements that vary in direction with the location of light stimulation in the SGI and increase in amplitude with progressively longer light durations. Therefore, these SC neurons can be defined as premotor and likely correspond to the premotor tectoreticular neurons identified in other species (Rodgers et al., 2006; Guitton and Munoz 1991; Munoz et al., 1991; Munoz and Guitton 1991; Olivier et al., 1993).

To relate to previous studies of CD, we focused our study on the centrolateral (CL) nucleus of the thalamus, an area surrounding the lateral borders of the MD nucleus which can be defined by its innervation from the deep layers of the SC and the substantia nigra pars reticulata (Krout et al., 2001), as well as its projections to the striatum (Tervo et al., 2016). We utilized the Pitx2-Cre line as well as a line which expresses Cre-recombinase in layer 5 cortical neurons (Rbp4-Cre, Gerfen et al., 2013) to examine CL connections with the SC and the motor cortex. Using a variety of viral tracing techniques, electron microscopy, and *in*-*vitro* dual opsin optogenetics, we examined the functional convergence of cortical and subcortical premotor circuits at the single cell level, providing insight into the thalamic contributions to gaze control.

## Materials and Methods

### Animals

All breeding and experimental procedures were approved by the University of Louisville Institutional Animal Care and Use Committee. Experiments were carried out using mice, of either sex, of C57BL/6J (RRID:IMSR Cat# JAX_000664), Pitx2-Cre (a line in which Pitx2 transcription factor expressing neurons express Cre-recombinase (Liu et al., 2002; generous donation by James F Martin, Baylor College of Medicine), Pitx2-Cre crossed with Ai9 (B6.Cg-Gt(ROSA) 26S ^ortm9 (*CAG-tdTomato) Hze/J*^ , a reporter mouse line expressing TdTomato in a Cre-dependent manner; RRID:IMSR_JAX:007909), Pitx2-Cre crossed with Ai32 (B6;129S-*Gt(ROSA)26Sor*^tm32(CAG-COP4*H134R/EYFP)Hze^/J, a reporter line expressing channelrhodopsin, ChR2, and enhanced yellow fluorescent protein, EYFP, in a Cre-dependent manner; RRID:IMSR_JAX:012569), or Rbp4-Cre in which Cre-recombinase is expressed in layer 5 cells in the cortex (RRID:MGI 4367067; Gerfen et al., 2013).

### Adeno-associated virus (AAV) injections for anatomy

To place intracranial virus injections for subsequent anatomical characterization, mice were deeply anesthetized with a mixture of ketamine (100–150 mg/kg) and xylazine (10–15 mg/kg). The analgesic meloxicam (1–2 mg/kg) was also administered prior to the surgery, and the anesthetic bupivacaine (3g/kg) was injected in the skin of the scalp. The eyes were covered with a lubricant ointment, and the animals were placed in a stereotaxic apparatus (Angle Two Stereotaxic, Leica, Wetzlar, Germany). An incision was made along the scalp, and a small hole was drilled in the skull overlying the left or right superior colliculus (SC), centrolateral nucleus of the thalamus (CL), or the primary and secondary motor cortex (together, MCtx). The viruses were delivered via a 34-gauge needle attached to a Nanofil syringe inserted in an ultramicropump. The needle was lowered vertically into the stratum griseum intermediale (SGI) of the SC (4.40mm posterior, 1.06mm lateral, and 1.84mm ventral to the Bregma), the CL (1.32mm posterior, 0.51mm lateral, 3.55mm ventral to Bregma), or MCtx (0.87mm posterior, 1.51mm lateral, 1.47 ventral to Bregma) to deliver volumes of 200– 400nL at a rate of 30nL/minute. Two minutes after the injections, the needle was removed, and the scalp skin was sealed with tissue adhesive (n-butyl cyanoacrylate). The animals were then placed on a heating pad until mobile. After surgery, animals were carefully monitored for proper wound healing, and subcutaneous meloxicam (1–2 mg/kg) injections were administered once per day for 48 hours post-surgery.

To label projections from SC cells that express Pitx2, viruses were injected unilaterally into the SGI of adult (>postnatal day 35) Pitx2-Cre mice with either Ef1α-DIO-dAPEX2, plasmid # 117174, Addgene, packaged using AAV serotype 9, which expresses the peroxidase enzyme Apex2 in a Cre-dependent manner, or hSyn-DIO-EGFP, plasmid #50457, Addgene, packaged using AAV serotype 1, which expresses green fluorescent protein, GFP, in a Cre-dependent manner. To label projections of MCtx layer 5 (L5) cortical neurons, the same viruses described above were injected into MCtx of adult Rbp4-Cre mice.

To label projections from SC-recipient CL neurons, unilateral injections were placed in the SGI of BLK6 mice of a virus that is transported in a transsynaptic manner to express Cre-recombinase in postsynaptic neurons (pENN.AAV.hSyn1.WPRE.hGH). Subsequently, a virus was injected into the ipsilateral CL that expresses GFP in the presence, and TdTomato in the absence, of Cre-recombinase (pAAV-Ef1α-DO_DIO-Tdt_EGFP-WPRE). To visualize MCtx projections in relation to Pitx2-epressing neurons in the SGI, AAV1-hSyn-DIO-EGFP was injected into MCtx of Pitx2-Ai9 mice.

### Biotinylated Dextran Amine (BDA) injections

To label tectothalamic axon projections via anterograde transport, adult C57BL/6J mice were prepared and placed in a stereotaxic apparatus as described above. An incision was made along the scalp, and a small hole overlying the SC was drilled in the skull. A glass pipette (20–40mm tip diameter) containing a 5% solution of BDA (Molecular Probes) in saline was lowered into the SC (4.40 mm posterior, 1.06 mm lateral, and 1.35 mm ventral to the Bregma) and BDA was iontophoretically ejected using 3 μA continuous positive current for 20 minutes. After removal of the pipette, the scalp skin was sealed, and the animals treated and monitored as described above.

### Histology of tissue used for anatomical analyses

Two to three weeks following injection of tracers and/or viruses, mice were deeply anesthetized with ketamine (450mg/kg) and transcardially perfused with a fixative solution of 4% paraformaldehyde in 0.1M phosphate buffer (PB) pH 7.4, or 2% glutaraldehyde and 2% paraformaldehyde in PB. In each case, the brain was removed from the skull and 70 ¼m thick coronal sections were cut using a vibratome (Leica Microsystems, Buffalo Grove, IL). Sections that contained fluorescent labels were mounted on slides or additionally stained using antibodies against either DsRed (made in rabbit, Clontech, catalogue #632496, 1:1000; RRID: AB10013483), or GFP (made in rabbit, Millipore, catalogue # AB3080; RRID: AB91337). Following overnight incubation in the DsRed or GFP antibodies, the sections were incubated in a 1:100 dilution of goat-anti-rabbit antibodies that were directly conjugated to fluorescent compounds (Alexa fluor 488 or 546; Invitrogen, Carlsbad, CA). After rinsing in PB, the sections were then mounted on slides. All sections that contained fluorescent labels were imaged using a confocal microscope (Olympus FV1200BX61).

Sections that contained Apex2 were processed immediately after cutting to prevent loss of enzyme activity. The sections were washed with PB, then 0.1M Na-acetate buffer (AB) pH 6.0 and then placed in a nickel-enhanced 3,3’- diaminobenzidine (DAB) solution (prepared by dissolving two 10mg tablets of DAB in 25mL of deionized (DI) H_2_O and 25mL of 3% Nickel ammonium sulphate in AB, then filtering). The DAB reaction was initiated by adding 15uL of 30% hydrogen peroxide (H_2_O_2_) and the sections were reacted for 30–60 minutes at room temperature on a shaker. The sections were subsequently rinsed in AB and then PB, and either mounted on slides or prepared for electron microscopy as described below. Sections that contained BDA were incubated in an avidin-biotin complex (ABC) solution (Vector Laboratories Standard Elite ABC kit PK-6100), reacted with nickel-enhanced DAB as described above, and mounted on slides or prepared for electron microscopy.

### Preparation of tissue for electron microscopy

Sections that contained terminals labeled by the DAB reaction were postfixed in 4% osmium tetroxide, dehydrated in an ethyl alcohol series (50, 70, 95 100%), and infiltrated with Durcupan resin in a vacuum oven. The next day, the sections were flat embedded in Durcupan resin between two sheets of Aclar plastic (Ladd Research) and the resin was cured at 60° C for 48 hours. Durcupan-embedded sections were first examined with a light microscope to select areas for electron microscopic analysis. Selected areas were mounted on blocks, ultrathin sections (70–80 nm, silver-gray interference color) were cut using a diamond knife, and sections were collected on formvar-coated nickel slot grids. Selected sections were stained for the presence of GABA using a previously described post-embedding immunocytochemical protocol (Bickford et al., 2015; Chomsung et al., 2008; Day-Brown et al., 2010). Briefly, we used a 0.25μg/ml concentration of a rabbit polyclonal antibody against GABA (Sigma227 Aldrich, St. Louis, MO, catalogue #A2052, RRID: AB_477652) and the GABA antibody was tagged with a goat-anti-rabbit antibody conjugated to 15 nm colloidal gold particles (BBI Solutions USA, Madison, WI). The sections were air dried and stained with a 10% solution of uranyl acetate in methanol for 30 min. To further enhance the contrast between the labeled structures and the tissue we dipped the sections in a lead citrate solution for 2 minutes and washed thoroughly with DI water before examining with an electron microscope.

### Ultrastructural analyses of electron micrographs

For electron microscopic analysis of DAB-labeled terminals and their postsynaptic targets, ultrathin tissue sections were examined using an electron microscope (Hitachi HT 7700) and every labeled terminal involved in a synapse was imaged (n=262 for Pitx2, n=256 for L5-MCtx, n=221 for SC profiles). The pre- and postsynaptic profiles were characterized based on size (measured using Image J, RRID: nif-000–30467, Maxim DL © 5 software) and the density of overlying gold particles.

### AAV injections for *in*-*vitro* electrophysiology

To label and photoactivate Pitx2, SC, and/or MCtx terminals in the CL, mice were prepared and placed in a stereotaxic apparatus, and viruses were injected as described above (AAV injections for anatomy). To label and/or activate Pitx2 projections, we unilaterally injected the SGI of Pitx2-Ai9 mice ranging in age between p30 and p40 with AAV9-Ef1a-double floxed-hChR2 (H134R)-EYFP. WPRE-HGhpA (Addgene, plasmid# 20298). To label and/or activate both L5-MCtx and SC projections, we injected MCtx of Rbp4-Cre mice with Syn-Flex-rc [ChrimsonR-tdTomato] (plasmid# 62723, Addgene, packaged using AAV serotype 5); this was paired with a second injection of AAV9-hSyn-hChR2 (H134R)-EYFP in the SC in the same hemisphere. We made another set of injections where we injected the SC with pAAV9-Syn-Chrimson-tdT (plasmid# 59171, Addgene) and MCtx with AAV9-Ef1a-DIO-hChR2 (H134R)- EYFP.WPRE.hGH. Twelve to 14 days following virus injections mice were euthanized for *in*-*vitro* recordings as described below. Viral serotypes, plasmid number, and titers are listed in Table 3.

### Slice preparation and optogenetic stimulation

Pitx2-Ai32 mice or virus-injected mice used for *in-vitro* recordings ranged in age from p45-p70. Mice were deeply anesthetized with isoflurane and decapitated. The brain was removed from the head, chilled in cold slicing solution (in mM: 2.5 KCl, 26 NaHCO_3_, 2.5 KCl, 1.25 NaH_2_PO_4_, 10 MgCl_2_, 2 CaCl_2_, 234 sucrose, and 11 glucose) for 2 minutes, and quickly transferred onto a filter paper (Reeve Angel, catalogue# 5200–110, size 11.0 cm) to block the brain for subsequent sectioning. Coronal slices (300 μm thick) were cut in chilled slicing solution using a vibratome (Leica VT1000 S). The slices were transferred into an incubation solution of oxygenated (95%O_2_ + 5%CO_2_) artificial cerebrospinal fluid (aCSF) containing the following (in mM: 126 NaCl, 26 NaHCO_3_, 2.5 KCl, 1.25 NaH2PO_4_, 2 MgCl_2_, 2 CaCl_2_, and 10 glucose) for 30 min to 6 hours. Individual slices were transferred into a recording chamber, which was maintained at 32°C by an inline heater and continuously perfused with oxygenated aCSF (2.5 ml/min). Slices were stabilized by a slice anchor or harp (Warner Instruments, 64– 0252). Neurons and labeled terminals were visualized on an upright microscope (Olympus, BX51WI) equipped with both differential interference contrast optics and a filter set for visualizing YFP (Chroma 49002) or TdTomato (Chroma 49005) using a 4x or 60x water-immersion objective (Olympus) and a CCD camera. Recording electrodes were pulled from borosilicate glass capillaries (World Precision Instruments) by using a Model P-97 puller (Sutter Instruments Co., Novato, CA). The electrode tip resistance was 4–6 MΩ when filled with an intracellular K^+^- based internal solution containing the following (in mM): 117 K-gluconate, 13.0 KCl, 1 MgCl_2_, 0.07 CaCl_2_, 0.1 EGTA, 10 HEPES, 2 Na_2_-ATP, and 0.4 Na_2_-GTP, with pH adjusted to 7.3 with KOH and osmolarity 290–300mOsm. Biocytin (0.5%) was added to this intracellular solution to allow morphological reconstruction of the recorded neurons.

Whole-cell recordings were obtained from the CL and surrounding areas. For Pitx2-Ai32 mice or virus-injected Pitx2-Ai9 mice, cells within the Pitx2 termination zones in the CL were targeted. For SC and L5-MCtx injection experiments in Rbp4-Cre mice, CL cells within the SC and L5-MCtx termination zones were targeted for recording. Video images of the patched cell locations were recorded using the CCD camera.

Recordings were obtained with an Axon Instruments multiclamp 700B amplifier (Molecular Devices), and a Digidata 1440A was used to acquire electrophysiological signals, with holding and voltage measurements corrected to account for a junction potential of −11mV. The stimulation trigger was controlled by Clampex 11.03 software (Molecular Devices). The signals were sampled at 20 kHz, and data were analyzed offline using ClampFit 11.4.1 software. For photoactivation of Pitx2, SC, and L5-MCtx, light from a blue (Prizmatix UHP 460) and/or red (Prizmatix UHP 630) light-emitting diode was reflected into a 60X water-immersion objective. This produced spots of light onto the submerged slice with diameters of ~0.3 mm (at full power, blue light 107.2 mW/mm^2^, red light 228.7 mW/mm^2^). Pulse duration and frequency were under computer control. For repetitive stimulation of terminals, pulse durations of either 1 or 10ms were used at frequencies of 1Hz, 2Hz, 5Hz, 10Hz, and 20Hz. For voltage clamp recordings, currents were recorded at −65mV. Changes in responses were then calculated by comparing equivalent periods (0.5s) of activity in the presence or absence of blue or red light stimulation (1, 2, 5, 10, and 20Hz). Typically, the measurements were based on the average of three sweeps of stimulus presentations.

For simultaneous activation of L5-MCtx and SC terminals in Rbp4-Cre animals, the following light activation protocol was used: 500ms of 20Hz red light pulses (1ms duration), followed by 5s of no light, followed by 500ms of 20Hz blue light pulses (1ms duration), followed by 3s of no light, followed by 1.5s of continuous red light stimulation with simultaneous 500ms of 20Hz blue light pulses (1ms duration) during the last 500ms of the continuous red light stimulation.

### Morphological analysis of cells filled during electrophysiological recording

Following recording, slices were placed in a fixative solution of 4% paraformaldehyde in PB for at least 24 hours. The sections were then rinsed in PB and incubated overnight in a 1:1000 dilution of streptavidin-conjugated to Alexafluor-633 (Invitrogen, Carlsbad, CA) in PB containing 1% Triton X-100. The following day some of the slices were washed in PB, pre-incubated in 10% normal goat serum (NGS) in PB and then incubated overnight in a 1:500 dilution (0.5μg/ml) of a rabbit anti-DsRed antibody (Clontech Laboratories, Inc. Mountainview, CA, catalogue #632496, RRID:AB_10015246) or a rabbit anti-GFP antibody (Millipore, Billerica, MA, catalogue #AB3080, RRID:AB_91337) with 1% NGS. The following day the sections were rinsed in PB and incubated for 1 hour in a 1:100 dilution of a goat anti-rabbit antibody conjugated to Alexa fluor-546 or Alexa fluor-488 (Invitrogen). The sections were then rinsed in PB and mounted on slides to be imaged with a confocal microscope (Olympus FV1200BX61). Confocal images of each slice were aligned with outlines of the CL nucleus and biocytin-filled CL cells were plotted. For cells that were not recovered, their location was plotted using the CCD images of the patch pipette location.

To classify neurons recorded *in*-*vitro* by their morphological characteristics, each confocal-imaged, biocytin-filled cell was cropped according to their dendritic arbor spread in Photoshop (Adobe 2025). The cropped cell with soma and dendrites was selected using the ‘Magic Wand Tool’ by adjusting the tolerance. Dendritic pieces that were not automatically picked up by the magic wand tool were manually added using the ‘Quick Selection Tool’. Once fully selected, the cell was cut out from the background using the following: Select- Inverse- Edit- Cut. Using ‘Eraser’ tool, the image was cleaned up to make sure only the soma and the dendrites of the cell remained in the image. The image was saved as a .tif file. The files were uploaded in ImageJ (Fiji; RRID: SCR_003070) software to create a mask using the following steps: Image-Adjust>Threshold set up>Apply> Analyze>Convert to Mask. The masked image was saved as a .tif file.

### Scholl ring analysis

Scholl ring analysis was performed on the masked images using the following steps in ImageJ: Plugin- Neuroanatomy- Sholl- SholI analysis (image or tracing). The center and end radius of the Scholl rings for each cell were defined by a region of interest (ROI, straight line) drawn from the approximate center of the soma to the most distal point in the dendritic arbor. The analysis was performed using the following steps: Analyze-Tools- ROI manager- Add the ROI size to the list. The “start radius” for these analyses was set to 15 pixels, with a “step size” (Scholl ring size) of 10 pixels. The Scholl ring diagrams produced were then used to quantify the number of dendritic “crossings” in each of 8 radially organized sections around the center (defined by the ROI described above) of each cell. For the analysis of dendritic orientation, we used methods similar those used by Krahe et al., 2011. Briefly, the Scholl rings were divided into four quadrants by passing two lines through the center of the soma at right angles to one another. These axial lines delineating the quadrants of the Sholl rings were arranged to maximize the difference in dendritic intersections between the two axial planes. For a given cell, the number of dendritic intersections passing through each ring was counted and grouped by axial plane (a_1_ + a_2_ or b_1_ + b_2_). The ratio of dendritic intersections in one plane versus the other (minimum/maximum) was taken as an index of dendritic orientation (DO*i*). A DO*i* of 1.0 represents a cell with equal number of intersections in both axial planes, whereas a cell with intersections in just one plane would have a DO*i* of 0.

### Pixel analysis of terminal fields surrounding biocytin-filled cells

Pixels were analyzed in ImageJ from .tif files to determine the density of Pitx2 and motor cortex axons and terminals that surrounded each recorded neuron. We only analyzed regions that included the dendritic arbors of the recorded neurons. All confocal images were taken at the same magnification, resolution, laser power, and all image stacks were collected in 5 μm increments. We used the ImageJ ‘Threshold’ function to separate labeled axon and terminal pixels from the background. The color channels for each image were split into red, green, and blue windows. Images for each channel were binarized, and the histogram function was used to calculate the total number of pixels in that channel. To calculate the density of pixels per area, the total area of each image was calculated using the ‘Measure’ tool, and the density was calculated by dividing the total number of pixels in one channel by the area of the image.

### Experimental design and statistical analysis

The experiments, animal lines, animal numbers, software, and statistical tests are listed in Table 1.

## Results

### SC Pitx2 and motor cortex projections innervate common areas of the thalamus

**Figure 1:**
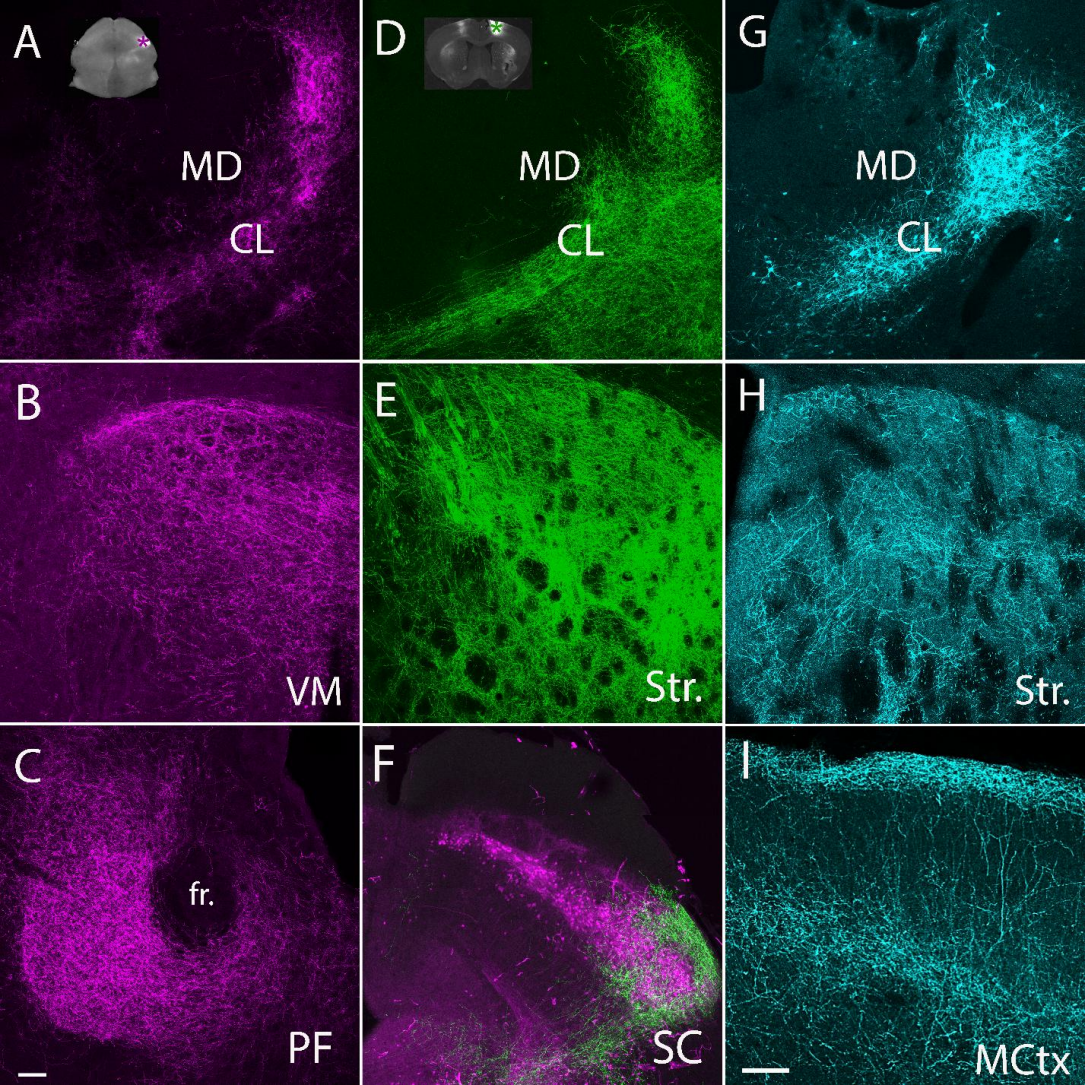
Pitx2, SC, and MCtx terminals innervate common structures. Unilateral Cre-dependent virus injections were placed in the SGI of Pitx2-Cre mice (A, inset, magenta asterisk) to express GFP in SC Pitx2 cells. Projections from Pitx2 cells (pseudocolored magenta) innervate the CL (A), VM (B), and PF (C), among other targets not shown. Unilateral non-Cre dependent virus injections were placed in primary and secondary motor cortex (MCtx) of Pitx2-Ai9 mice (D, inset, green asterisk, see also Figure S1A) to express GFP in MCtx neurons. Projections from MCtx are shown in green. The MCtx innervates the CL (D), striatum (Str., E), and SGI (F) where Pitx2 cells are located (shown in magenta), and other targets (not shown). A virus that is transported transsynaptically to express Cre-recombinase in postsynaptic neurons was injected in the SC of C57BL/6J mice. This was paired with a second injection of a Cre-dependent virus in the CL to express GFP in CL neurons innervated by the SC. SC-recipient CL cells (G, pseudocolored cyan) innervate multiple structures including the Str (H) and MCtx . SC: superior colliculus; SGI: stratum griseum intermediale; Str: Striatum; CL: centrolateral nucleus of the thalamus; VM: ventromedial nucleus of the thalamus; PF: parafascicular nucleus of the thalamus; MCtx: primary and secondary motor cortex; fr: fasciculus retroflexus. Scale bar in C:100μm, applies to panels A-H. Scale bar in I: 100μm.

We took advantage of the Pitx2-Cre mouse line (Masullo et al., 2019) to label and visualize ascending projections from premotor neurons of the SC. We injected a Cre-dependent virus in the SGI of Pitx2-Cre mice to induce the expression of GFP and found that SC Pitx2 cells project to a variety of thalamic nuclei including the centrolateral (CL), ventromedial (VM) and parafascicular (PF) nuclei (Figure 1A, B, C, magenta). To label descending projections from the motor cortex to the CL, we injected areas M1 and M2 (hereafter referred to as MCtx) in Pitx2-Ai9 mice with a non-Cre dependent virus to induce the expression of GFP (Figure 1D inset, Figure S1A). This resulted in the labeling of projections to the same thalamic regions innervated by Pitx2 cells, including the CL (Figure 1D, green), as well as the striatum (Figure E, green), and lateral SGI where the Pitx2 cells are located (Figure 1F, Figure S2B, MCtx projections green, Pitx2 cells magenta). We also observed a close association between terminals originating from Pitx2 and MCtx neurons in the CL (Figure S2A, Pitx2 terminals in magenta, MCtx terminals in green), suggesting potential convergence of ascending and descending motor inputs to CL neurons.

### SC recipient CL cells project to the MCtx and striatum

To examine the projections of CL cells that receive input from the SC, we injected the SC of C57BL/6J mice with a virus that is transported in a transsynaptic manner to express Cre-recombinase in postsynaptic neurons (Zingg et al., 2017). Subsequently, a second virus was injected into the ipsilateral CL to induce the expression of GFP in the presence of Cre-recombinase (Figure 1G, cyan). The labeled CL neurons projected to both the striatum (Figure 1H, cyan) and MCtx (Figure 1I, cyan, Figure S1B black stipple; CL terminals concentrated in layers 1 and 4 as previously described for thalamic input to the motor cortex, Bopp et al., 2017). Thus, MCtx and the premotor regions of the SC are interconnected via MCtx projections to the SGI as well as reciprocal connections with SC-recipient CL neurons.

### Comparison of the ultrastructure of SC Pitx2 and L5-MCtx presynaptic profiles

**Figure 2:**
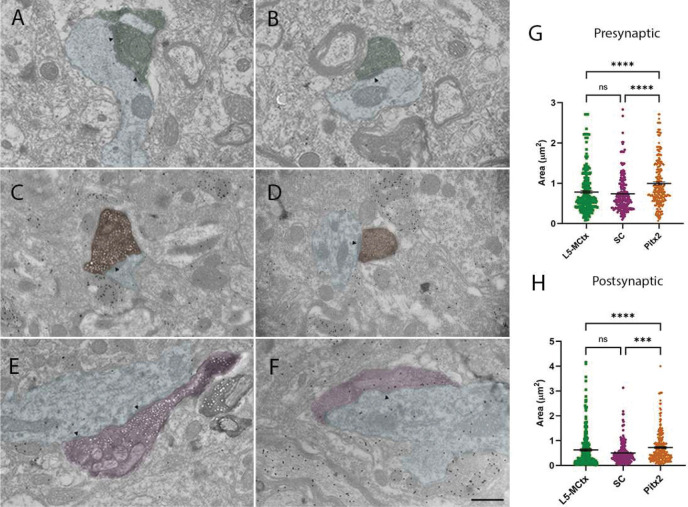
Ultrastructure of L5-MCtx, SC and Pitx2 terminals in the CL. Transmission electron micrographs illustrate L5-MCtx terminals in the CL labeled via Cre-dependent virus injections in MCtx of Rbp4-Cre mice (A,B green overlay), SC terminals in the CL labeled via biotinylated dextrin amine (BDA) injections in the SC of C57BL/6J mice (C,D orange overlay), and Pitx2 terminals in the CL labeled via Cre-dependent virus injections in the SC of Pitx2-Cre mice (E,F, magenta overlay). Presynaptic terminals were revealed with 3,3’- diaminobenzidine (DAB, dark reaction product) and sections were stained to reveal GABA using an antibody tagged with gold particles. Black arrowheads indicate postsynaptic densities at synapses and blue overlays indicate postsynaptic dendrites. Statistical comparison of L5-MCtx (n=256), SC (n=221), and Pitx2 (n=262) terminal profiles in the CL revealed that Pitx2 terminals are significantly larger than both L5-MCtx (p<0.0001) and SC (p<0.0001) terminals (G). Comparison of the postsynaptic dendritic partners of the three types of terminals revealed Pitx2 terminals contact dendrites that are significantly larger than those contacted by L5-MCtx (p<0.0001) or SC (p<0.0001) terminals (H). Scale bars: 600nm applies to A-F. Shown are mean and standard error of the mean (G, H). Kruskal- Wallis test was used for statistical analysis.

To examine the ultrastructure of synaptic terminals in the CL, we injected the SC of C57BL/6J mice with the tracer biotinylated dextran amine (BDA) or injected a Cre-dependent virus in the SC of Pitx2-Cre mice to induce the expression of Apex2 in Pitx2 neurons. In addition, we injected a Cre-dependent virus in the MCtx of Rbp4-Cre mice to induce the expression of Apex2 in layer 5 neurons (L5-MCtx). In all cases, tissues were reacted with 3,3- diaminobenzidine (DAB, dark homogeneous precipitate) to reveal the tracer- or virus-labeled terminals and additionally stained with an antibody against GABA tagged with gold particles (small dark dots in electron micrographs). We identified synapses by the presence of a postsynaptic density and synaptic cleft and measured the sizes of the presynaptic terminals (L5-MCtx, n=256, SC n=221, and Pitx2, n=262) and their postsynaptic dendrites (Figure 2A–F).

The density of gold particles overlying each pre- and/or postsynaptic profile was used to determine whether they contained GABA. The average gold particle density overlying Pitx2 terminals was 9 ± 7.26 gold particles/μm^2^, and L5-MCtx terminals was 5 ± 4 gold particles/μm^2^. Since Pitx2-Cre and Rbp4-Cre lines label glutamatergic cells only (Masullo et al., 2019; Callaway et al., 2021), we used the average + 2X the standard deviation of the gold particle density overlying Pitx2 terminals (23.52) as the cutoff value for identifying GABAergic (>24 gold particles/μm^2^) vs non-GABAergic (<24 gold particles/μm^2^) profiles in the CL (Figure S3E). Occasionally, L5-MCtx, SC or Pitx2 terminals contacted postsynaptic dendrites that were identified as GABAergic (L5-MCtx n=1, SC n=3, Pitx2 n=1). We also found some BDA-labeled SC terminals (n=43) that were identified as GABAergic (Figure S3D, F, G). Tracer injections could have labeled fibers of passage and/or inputs that branch to innervate the SC and CL. Since GABAergic projections from the SC primarily target regions outside the thalamus (Whyland et al., 2020), these GABAergic tracer-labeled terminals could potentially represent projections from substantia nigra neurons that branch to innervate the SC and thalamus (Anderson and Yoshida 1980; Beckstead 1983). Therefore, these GABAergic terminals were excluded from further analysis.

Quantitative analysis revealed that in the CL, Pitx2 terminals are significantly larger than L5-MCtx terminals (p<0.001) as well as the nonGABAergic SC terminals (p<0.0001) (Figure 2G; Kruskal-Wallis test). Likewise, dendrites postsynaptic to Pitx2 terminals were larger than those contacted by L5-MCtx terminals (p<0.0001, Kruskal-Wallis test) or SC terminals (p<0.0001; Figure 2H; Kruskal-Wallis test). No significant difference was found between L5-MCtx and SC presynaptic terminals and their postsynaptic dendrites (p>0.9999 in both cases, Kruskal-Wallis test).

### Photoactivation of Pitx2 terminals in CL

**Figure 3:**
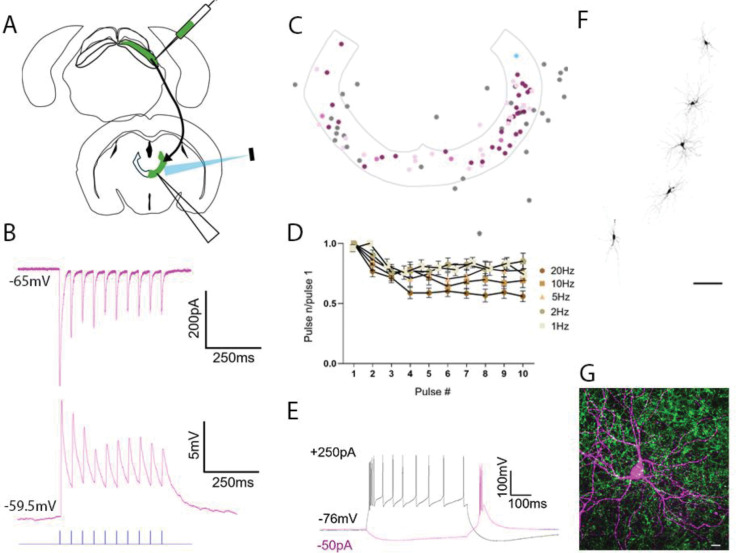
Synaptic properties of Pitx2 projections to the CL. *In-vitro* whole cell patch clamp recordings were obtained from a total of 116 CL cells in Pitx2-Ai9 mice with Cre-dependent SC virus injections to express channelrhodopsin in Pitx2 neurons (n=6) or in Pitx2-Ai32 mice (n=4). Of these, 55 cells responded to optogenetic activation of Pitx2 terminals. A) A schematic of SC injection site and recording site in the CL of Pitx2-Ai9 mice is shown. Recording locations were the same in Pitx2-Ai32 mice. B) Example responses of a CL cell to photoactivation of Pitx2 terminals (1ms blue light pulses at 20Hz, indicated by blue ticks) recorded in voltage clamp (top) and current clamp (bottom). C) Schematic of the location of biocytin-filled cells in the CL. Magenta dots: cells with strong responses (n=30), light pink dots: cells with moderate responses (n=25); grey dots: non-responsive cells, blue dot: cell with responses illustrated in B. D) Population average of response amplitudes to each light pulse in 1, 2, 5,10 and 20Hz trains divided by the response amplitude to the first light pulse of each train (for CL cells that responded strongly or moderately, n=55). CL responses to photoactivation of Pitx2 terminals exhibited frequency-dependent depression. E) Injection of positive current steps (+250pA) in Pitx2-responsive CL neurons elicited trains of action potentials (black trace) while injection of negative current steps (−50pA) induced rebound burst firing (magenta trace). F) Confocal image of biocytin filled CL cells imaged post-recording (scale bar: 100μm). G) Confocal image of a biocytin filled CL cell (magenta) surrounded by Pitx2 terminals (green, scale bar: 10μm). by Pitx2 terminals (green, scale bar: 10μm).

To determine how activation of Pitx2 terminals affects neurons in the CL, we induced the expression of the blue light-activated opsin Channelrhodopsin (ChR2) and the fluorophore GFP by either crossing Pitx2-Cre mice with a reporter line (Ai32) or by injecting a Cre-dependent virus in the SC of Pitx2-Ai9 mice (Figure 3A). We then recorded from neurons in and around the CL nucleus in voltage clamp (Figure 3B, top) or current clamp (Figure 3B, bottom) in acute coronal slice preparations maintained *in-vitro* while activating Pitx2 terminals with 10 consecutive blue light pulses (1ms duration at 1, 2, 5, 10 and 20Hz). We added biocytin to the internal solution in our recording pipettes to subsequently examine the morphology of the recorded neurons and plot their locations on templates of the CL. We recorded from 68 CL neurons in virus injected mice and 48 CL neurons in Pitx2-Ai32 mice (Table 2). Of those, 55 cells CL neurons responded to photoactivation of Pitx2 terminals with excitatory postsynaptic currents (EPSCs) that exhibited amplitudes >10pA. We defined responses with amplitudes >100pA as strong (n=30, virus-injected, n=19, Ai32, n=11) and those with amplitudes of 10–100pA as moderate (n=25, virus-injected, n=18, Ai32, n=7). The average peak amplitudes were 247.59±271.97 in virus injected animals (n=6) and 221.13±214.56 in Ai32 mice (n=4). Photoactivation of Pitx2 terminals occasionally generated action potentials in postsynaptic CL neurons (Figure S4A, n=0 in virus injected mice, n=4 in Ai32 mice) and these were excluded from subsequent analyses of frequency-dependency and comparisons of response amplitudes.

We identified the location of the recorded CL cells based on recovered biocytin-filled cells (Figure 3F) and/or photos of the location of the recording pipettes. We then plotted the location of these cells in a schematic of the horseshoe-like extent of the CL nucleus (borders defined based on the projections of SC Pitx2 projections; Figure 3C). Cells outside of the CL did not respond to Pitx2 photoactivation (grey dots). Cells that responded strongly (magenta and blue dots) or moderately (pink dots) were located within the CL.

CL responses to photoactivation of Pitx2 terminals exhibited frequency-dependent depression. For CL cells that responded either strongly or moderately, the amplitudes of responses to each light pulse in 1, 2, 5,10 and 20Hz trains were divided by the amplitude of the response to the first light pulse of each train to calculate a ratio. The population average of these ratios is plotted in Figure 3D which demonstrates that the amplitudes of responses to the 2nd-10th pulse decreased relative to the amplitude of responses to the first pulse, and that this synaptic depression increased with train frequency.

When the CL cells were sufficiently depolarized (via injection of positive current steps), trains of action potentials elicited “tonic firing” (Figure 3E, black trace), while sufficient hyperpolarization (via injection of negative current steps) induced rebound “burst firing” (Figure 3E, magenta trace). Thus, CL neurons display the voltage-dependent intrinsic membrane properties of typical thalamic relay neurons (Sherman, 2001). Finally, confocal images were taken for subsequent analysis of the morphology of recorded neurons and the density of the surrounding terminals. The close association between the dendritic arbors of recorded neurons and surrounding Pitx2 terminals is illustrated in Figure 3G.

### Photoactivation of L5-MCtx terminals in CL

**Figure 4:**
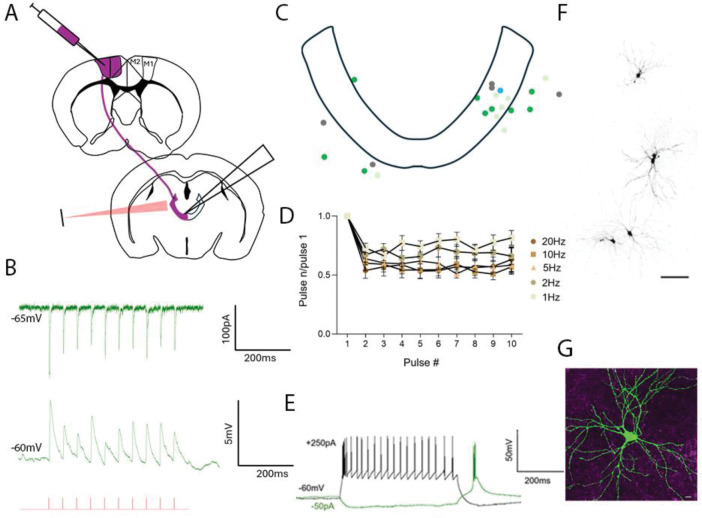
Synaptic properties of L5-MCtx projections to the CL. *In-vitro* whole cell patch clamp recordings were obtained from a total of 111 CL cells in Rbp4-Cre mice that received virus injections in the MCtx to express Chrimson or ChR2 in layer 5 neurons. Of those, 23 CL cells responded to optogenetic activation of L5-MCtx terminals. A) A schematic of injection sites in MCtx and recording sites in the CL of Rbp4-Cre mice is shown. B. Example responses (green) of a CL cell to photoactivation of L5-MCtx terminals (1ms red or blue light pulses at 20Hz, indicated by red ticks) recorded in voltage clamp (top) and current clamp (bottom). C) Schematic of the location of biocytin-filled cells in the CL that responded to MCtx input only (16 recovered). Dark and light green dots: cells with strong and moderate responses, respectively; grey dots: non-responsive cells, blue dot: cell with responses illustrated in B. D) Population average of response amplitude to each light pulse in 1, 2, 5,10 and 20Hz trains are divided by the response amplitude to the first light pulse of each train (n=18). On average, CL responses to photoactivation of L5-MCtx terminals exhibited frequency-dependent depression. E) Injection of positive current steps (+250pA) in L5-MCtx-responsive CL neurons elicited trains of action potentials (black trace) while injection of negative current steps (−50pA) induced rebound burst firing (green trace). F) Confocal image of biocytin filled CL cells imaged post-recording (scale bar: 100μm). G) Confocal image of a biocytin filled CL cell (green) surrounded by L5-MCtx terminals (magenta, scale bar: 10μm).

Since the CL receives projections from both the MCtx and SC, the goal of the next set of experiments was to determine how activation of L5-MCtx terminals affected neurons in the CL. Cre-dependent virus injections were placed in the MCtx of Rbp4-Cre mice to induce the expression of either ChR2 and GFP, or the red-shifted opsin Chrimson and the fluorophore TdTomato (Figure 4A). In some mice (n=9, Table 2), these injections were paired with non-Cre dependent virus injections in the SC to induce the expression of complementary opsins and fluorophores (Chrimson/TdTomato or ChR2/GFP) to examine photoactivation of SC terminals and possible convergence of MCtx and SC inputs (described below in subsequent sections). We recorded from cells in and around the CL nucleus in voltage clamp (Figure 4B, top) or current clamp (Figure 4B, bottom) while activating L5-MCtx terminals with 10 consecutive blue and/or red-light pulses (1ms duration at 1, 2, 5, 10 and 20Hz). We recorded from 111 CL neurons (Table 2) and 23 responded to photoactivation of L5-MCtx terminals with average peak amplitudes of 132.75±143.91 (Chrimson n=6) and 90.77±66.30 (ChR2 n=17). Photoactivation of L5-MCtx terminals with either opsin occasionally generated action potentials in postsynaptic CL neurons (Figure S4B, n=3) and these were excluded from subsequent analyses of frequency-dependency and comparison of response amplitudes.

We identified the location of the recorded CL cells based on recovered biocytin-filled cells (Figure 4F) and/or photos of the location of the recording pipettes and plotted these locations in a schematic of the CL nucleus (Figure 4C). CL cells with strong (n=8) and moderate (n=15) responses to photoactivation of L5-MCtx terminals were located both within and outside of the CL (dark and light green dots, respectively). The locations of a few non-responsive cells are shown as well (grey dots).

Similar to CL responses to photoactivation of Pitx2 terminals, CL responses to photoactivation of L5-MCtx terminals exhibited frequency-dependent depression. For CL cells that responded strongly or moderately to every light pulse at 1, 2, 5, 10, and 20Hz (n=18) the ratios of the response amplitudes relative to the first pulse of each train were calculated and the population average of these ratios plotted (Figure 4D). This demonstrates that the amplitudes of responses to the 2ndpulse decreased relative to the amplitude of responses to the first pulse, and that this depression increased with train frequency.

In addition, CL neurons that responded to photoactivation of L5-MCtx terminals also exhibited tonic (black trace) and burst (green trace) firing (Figure 4E). Finally, the close association between the dendritic arbors of recorded neurons and surrounding L5-MCtx terminals is illustrated in Figure 4G. A non-linear regression analysis with curve fit revealed a trend for increased density of surrounding L5-MCtx terminals eliciting larger amplitude responses in CL neurons (Figure S5A).

### Comparison of CL responses to photoactivation of L5-MCtx, SC, and Pitx2 terminals

**Figure 5:**
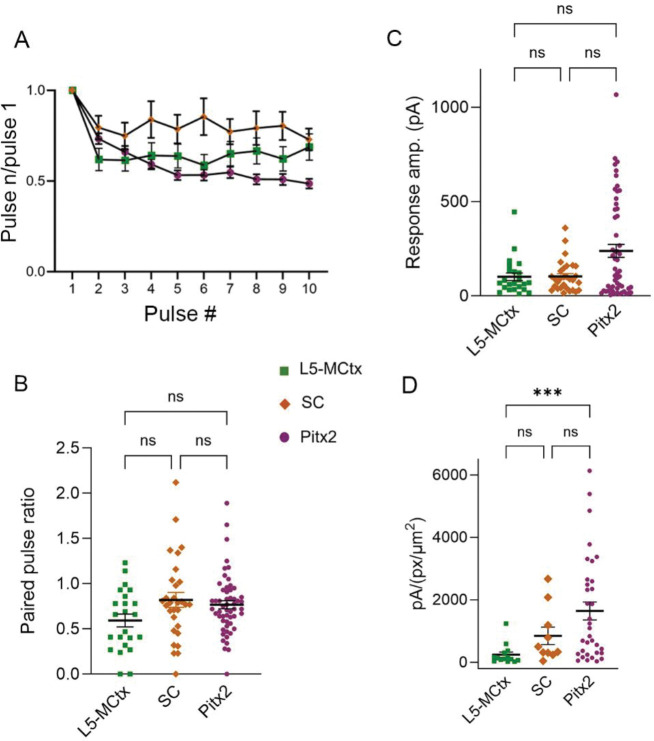
Comparison of synaptic properties of L5-MCtx, SC, and Pitx2 projections to the CL. A) Population average of response amplitudes to photoactivation of L5-MCtx (green squares), SC (orange diamonds) and Pitx2 (magenta dots) for each light pulse in 20Hz trains divided by the response amplitude to the first light pulse of each train (for CL cells that responded to each input, L5-MCtx, n=23, SC n=30, Pitx2, n=55. B) The paired pulse ratio (response amplitudes to pulses 2 of a 20Hz train divided by the amplitude of the response to pulse 1) for each CL cell that responded strongly or moderately to the photoactivation of L5-MCtx (n=23), SC (n=30) or Pitx2 (n=55) terminals. We found no significant difference in the frequency-dependence of L5-MCtx versus SC (p=0.1722), L5-MCtx vs Pitx2 (p=0.2197), or SC vs Pitx2 (p>0.9999) responses. C) The amplitudes of CL responses to photoactivation of L5-MCtx, SC, and Pitx2 terminals were not significantly different from each other (L5-MCtx vs Pitx2, p=0.4792; L5-MCtx vs SC, p>0.9999; SC vs Pitx2, p=0.7446). D). Response amplitudes plotted as a function of the density of the surrounding L5-MCtx (green), SC (orange) or Pitx2 terminals (magenta) that were photoactivated (measured as pixel density, see text). There was a significant difference (p=0.0005) in response amplitude per terminal density between CL neurons receiving L5-MCtx vs Pitx2 input. There was no significant difference in the response amplitude per terminal density for CL neurons receiving L5-MCtx vs SC input (p=0.1055) or SC vs Pitx2 input (p>0.9999). Shown are mean and the standard errors of the mean (B, C, D). All tests of significance were carried out using Kruskal-Wallis.

Similar experiments were carried out to examine how photoactivation of SC terminals affects neurons in the CL. We recorded from a total of 118 CL cells (Table 2) of which 30 cells responded to photoactivation of SC terminals with either strong (n=13) or moderate (n=17) EPSC amplitudes. For cells that responded to each input (L5-MCtx, n=23, SC, n=30, Pitx2, n=55), the ratios of the response amplitudes relative to the first pulse of each train were calculated and the population average of these ratios plotted (Figure 5A). We also calculated the paired pulse ratio (response amplitude to pulse 2 divided by response amplitude to pulse 1). The ratios for most of the CL cells that responded strongly or moderately to Pitx2 (magenta dots), L5-MCtx (green squares), or SC (orange diamonds) photoactivation are plotted in Figure 5B. Pitx2 responses exhibited frequency-dependent depression that was not significantly different from SC (p>0.9999, Kruskal-Wallis test) or L5-MCtx (p=0.2197, Kruskal-Wallis test) responses. The frequency-dependent depression of L5-MCtx versus SC responses was also not significantly different (p=0.1722, Kruskal-Wallis test).

Next, we compared the peak response amplitudes to photoactivation of each terminal type. Photoactivation of Pitx2 terminals elicited responses that were not significantly different from the responses of L5-MCtx (p=0.4792, Kruskal-Wallis test) or SC (p=0.7446, Kruskal-Wallis test) terminal photoactivation (Figure 5C). However, when we analyzed the amplitudes of responses to photoactivation of the 3 terminal types as a function of the density of the surrounding terminals (calculated as pixel density, Figure 5D), we found the amplitude per pixel density (pA/(px/um^2^)) of Pitx2 responses were significantly larger (p=0.0005, Kruskal-Wallis test) than those of L5-MCtx responses. However, there was no significant difference between these measures for L5-MCtx versus SC responses (p=0.1055, Kruskal-Wallis test) or SC versus Pitx2 responses (p>0.9999, Kruskal-Wallis test).

### CL neurons receive convergent inputs from SC and L5-MCtx

**Figure 6:**
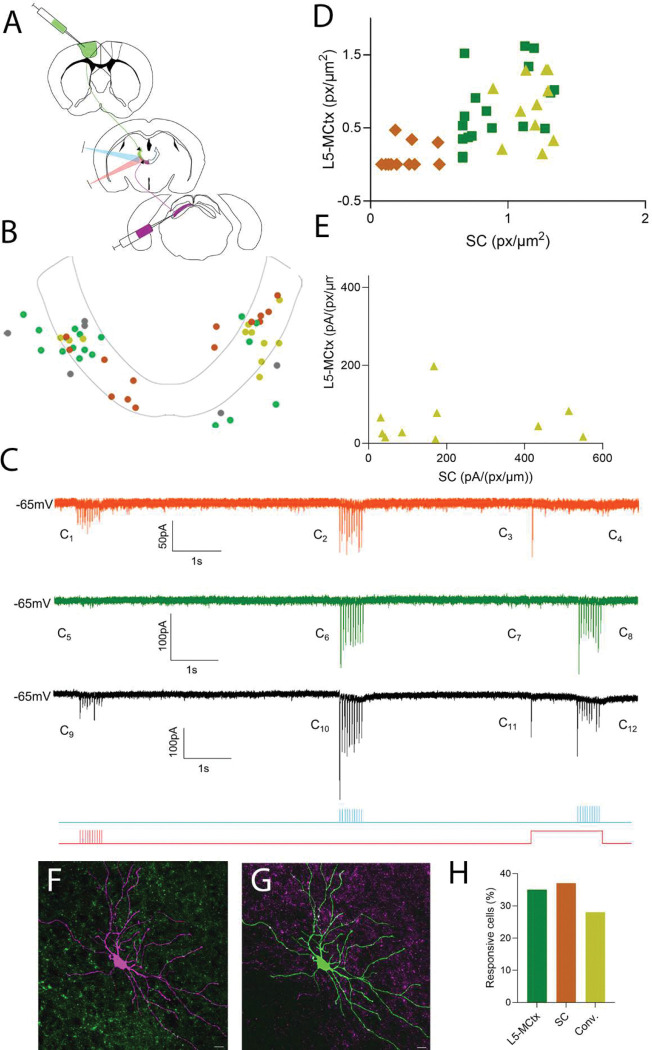
Convergent SC and L5-MCtx input to individual CL neurons. *In-vitro* whole cell patch clamp recordings were obtained from a total of 101 CL cells in Rbp4-Cre mice that received Cre-dependent virus injections in MCtx to express Chrimson or ChR2 in layer 5 neurons and non-Cre dependent virus injections in the SC to express ChR2 or Chrimson in SC neurons. Of those, 19 CL cells responded to optogenetic activation of L5-MCtx terminals only, 20 to SC only, and 15 to both L5-MCtx and SC terminals. A) A schematic of the injection and recording sites is shown. B) Schematic of the location of biocytin filled CL cells that responded to photoactivation of L5-MCtx terminals only (green dots), SC terminals only (orange dots), both L5-MCtx and SC terminals (convergent, mustard dots), and non-responsive cells (gray dots). C) Example traces of CL cells that responded to photostimulation of SC terminals that expressed Chrimson (orange), L5-MCtx terminals that expressed ChR2 (green), or both L5-MCtx ChR2 expressing terminals and SC Chrimson expressing terminals (black). The light protocol is shown below the traces. Chrimson-expressing terminals can be activated by both red and blue light pulses (**C**_**9**_**, C**_**12**_). Chrimson responses to blue light pulses can be blocked by using an “occlusion protocol”. The occlusion protocol consists of a continuous 2 second pulse of red light, which after an initial release of the neurotransmitter from the Chrimson-expressing terminals (**C**_**3**_) blocks any responses to simultaneous blue light pulses (**C**_**4**_). ChR2-expressing terminals do not respond to red-light (**C**_**5,**_ **C**_**7**_) but do respond to blue light pulses (**C**_**6**_) even in the presence of the long red-light pulse (**C**_**8**_). Thus, using this paradigm in dual opsin experiments allowed us to examine responses of the same cells to terminals expressing Chrimson (**C**_**9,**_ **C**_**11**_), ChR2 (**C**_**12**_), or both (**C**_**10**_). D) Comparison of the pixel density of L5-MCtx and SC terminal fields for cells that responded to SC only (orange diamonds), L5-MCtx only (green squares), or both L5-MCtx and SC (convergent, mustard triangles). E) L5-MCtx and SC response amplitudes per pixel density of surrounding photoactivated terminals for CL cells receiving convergent input. F and G: Confocal images of a CL neuron that received convergent input. CL cell is shown in magenta and L5-MCtx projections are shown in green (F). The same cell is shown in green with SC projections in magenta (G). Scale bar = 10μm. H) Percentage of total responsive CL cells (n=54) that responded to photoactivation of L5-MCtx (35%), SC (37%), or both (28%) terminals. See table 2 for reference.

Our next question was whether there is functional convergence of ascending and descending motor inputs to individual CL neurons. As described above, these experiments were carried out in slices prepared from Rbp4-Cre mice that received virus injections to express complementary opsins and fluorophores in MCtx layer 5 neurons and in SC neurons (Figure 6A). Chrimson-expressing terminals can be activated by both red and blue light pulses (Figure 6C_9_–C_12_). However, Chrimson responses to blue light pulses can be blocked by using an “occlusion protocol” (Masterson et al., 2025). The occlusion protocol consists of a continuous 2 second pulse of red light, which after an initial release of the neurotransmitter from the Chrimson-expressing terminals (Figure 6C_3_) blocks any responses to simultaneous blue light pulses (Figure 6C_4_). ChR2-expressing terminals do not respond to the red-light pulses (Figure 6C_5_ and C_7_) but do respond to blue light pulses (Figure 6C_6_) even in the presence of the long red-light pulse (Figure 6C_8_). Thus, using this paradigm in dual opsin experiments allowed us to examine responses of the same cells to terminals expressing Chrimson (Figure 6C), ChR2 (6C_12_) or both (6C_10_). We recorded from 101 CL neurons in Rbp4-Cre mice (n=9) where there was good expression of complementary opsins in both L5-MCtx and SC terminals (Table 2). Of these, 55 cells were responsive - 19 (35%) responded only to L5-MCtx inputs (Figure 6H, green bar), 20 (37%) responded only to SC inputs (Figure 6H, orange bar), and 15 (28%) responded to both SC and L5-MCtx inputs (Figure 6H, mustard bar). In other words, of the 35 CL neurons that responded to SC, 43% also responded to L5-MCtx input and of the 34 CL cells that responded to L5-MCtx input, 44% also responded to SC input.

As described above, we added biocytin in our recording pipettes to subsequently examine the morphology of the recorded neurons and plot their locations on templates of the CL (Figure 6B, orange dots responsive to SC only, green dots responsive to L5-MCtx only, mustard dots responsive to both SC and L5-MCtx, grey dots unresponsive to either input). To determine whether terminal density was related to the recorded responses, we measured the total number of pixels (px) and pixel densities (px/μm^2^) of SC and L5-MCtx terminal fields surrounding the recorded CL cells (Figure 6D). Example terminal fields surrounding a labeled CL neuron are shown in Figure 6F (L5-MCtx terminals green, CL cell magenta) and Figure 6G (SC terminals magenta, CL cell green). We then calculated the response amplitude per pixel density (pA/(px/μm^2^)) for CL cells that received convergent input. In most cases, CL neurons that received convergent inputs responded more strongly to SC than L5-MCtx when terminal density was taken into account (Figure 6E).

We also compared the densities of L5-MCtx and SC pixel densities surrounding CL cells that responded to L5-MCtx only, SC only, or both L5-MCtx and SC. We found a significant difference (p=0.0006, Kruskal-Wallis test) in pixel densities surrounding CL cells that responded to photoactivation of L5-MCtx only or SC only. There was also a significant difference (p=0.0122, Kruskal-Wallis test) in SC pixel densities surrounding the CL cells that responded to L5-MCtx only and L5-MCtx+SC (convergent, Figure S5B and C).

### Morphological analysis of the CL neurons

**Figure 7:**
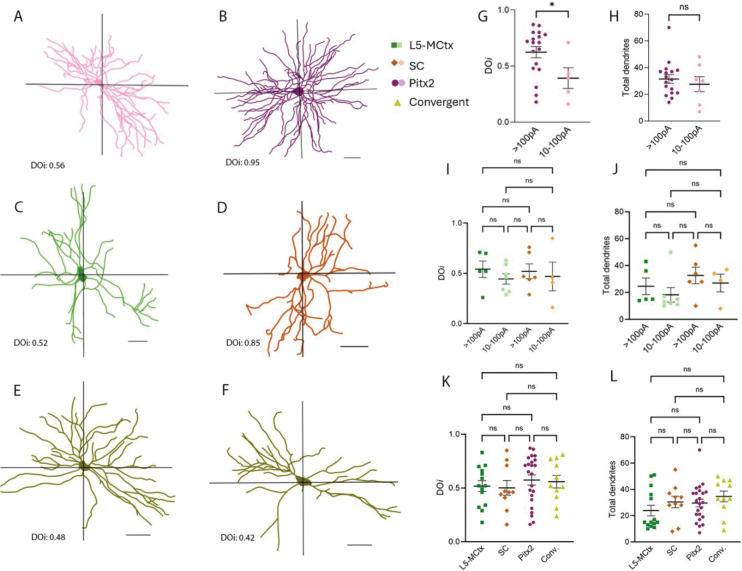
Morphology of CL cells that responded to photoactivation of Pitx2, L5-MCtx and/or SC terminals. A-F) Examples of the morphologies of CL neurons that responded strongly (>100pA, A) or moderately (10–100pA, B) to photoactivation of Pitx2 terminals, to photoactivation of L5-MCtx terminals (C), SC terminals (D), or both L5-MCtxband SC terminals (E, F). For all cells, a dendritic orientation index (DO*i*) was calculated using methods described in Krahe et al., 2011. CL cells with strong responses to photoactivation of Pitx2 terminals exhibited a larger number of dendritic crossings across two axial planes (higher DO*i*) when compared to moderate responders (p=0.0360, G) but there was no significant difference in the total number of dendrites in these two groups (p=0.8839, H). I) No significant difference was found in the dendritic orientation (DO*i*) of CL cells that responded strongly or moderately upon photoactivation of L5-MCtx terminals (strong vs moderate: p>0.9999) or SC terminals (strong vs moderate: p>0.9999). J) No significant difference was found in the total number of dendrites of CL cells that responded strongly or moderately upon photoactivation of L5-MCtx or SC terminals; p>0.9999 in each case. K) There was no significant difference in the DO*i* of CL cells that responded to photoactivation of L5-MCtx, SC, Pitx2, or both L5-MCtx and SC (conv.) inputs (p>0.9999 for all comparisons). L) There was no significant difference in the total dendrites of CL cells that responded to photoactivation of L5-MCtx, SC, Pitx2, or both L5-MCtx and SC (conv.) inputs (p>0.9999 for every comparison except L5-MCtx vs convergent input receiving CL cells, p=0.3703). Scale bars in A-F: 50μm. Mann-Whitney nonparametric t-test was used in G and H. Kruskal-Wallis test was used in I-L.

To quantify the morphology of biocytin-filled CL cells that responded to photoactivation of SC Pitx2 terminals, we traced the filled cells and calculated dendritic orientation indices (DO*i*) using methods described in Krahe et al., 2011. This index is lower for cells with oriented arbors (Figure 7A) and higher for cells with symmetrical arbors (Figure 7B). We then compared the DO*i*’s of cells that responded strongly or moderately to photoactivation of Pitx2 input and found that cells with strong responses exhibited significantly higher DO*i*’s compared to cells with moderate responses (p=0.0360, Mann-Whitney test, Figure 7G). However, the total number of dendrites of these two groups were not significantly different from each other (p=0.8839, Mann-Whitney test). There also was no consistent relationship between the DO*i*’s or response strength with location in the CL (Figure S6).

We applied the same methods to biocytin-filled CL cells that received only L5-MCtx input or only SC input to compare the DO*i* and total number of dendrites. We found no significant difference in these measures in CL neurons that responded strongly or moderately to photoactivation of L5-MCtx terminals (dendritic orientation index, p>0.9999, Kruskal-Wallis test, total dendrites, p>0.9999, Kruskal-Wallis test) and strongly or moderately to photoactivation of SC terminals (dendritic orientation index, p>0.9999, Kruskal-Wallis test, total dendrites, p>0.9999, Kruskal-Wallis test). In addition, no significant difference was found in the DO*i* (p>0.9999 for all comparisons, Kruskal-Wallis, Figure 7K) or total dendrites (p>0.9999 for every comparison except L5-MCtx vs convergent input, p=0.3703, Figure 7L) of CL neurons that responded to photoactivation of L5-MCtx, SC, both L5-MCtx+SC, or Pitx2 terminals.

## Discussion

While cortical and subcortical interactions occur across multiple brain regions and levels, the thalamus should be considered a key player, as all thalamic nuclei are reciprocally connected to the cortex (Clasca et al., 2012). Likewise, the superior colliculus (SC) receives input from most regions of the cortex and is a major source of ascending input to the thalamus (Harting et al., 1980, 1992, 2001). Therefore, the tectorecipient thalamus is likely a vital participant in the dynamic interplay between cortical and subcortical activity patterns.

**Figure 8:**
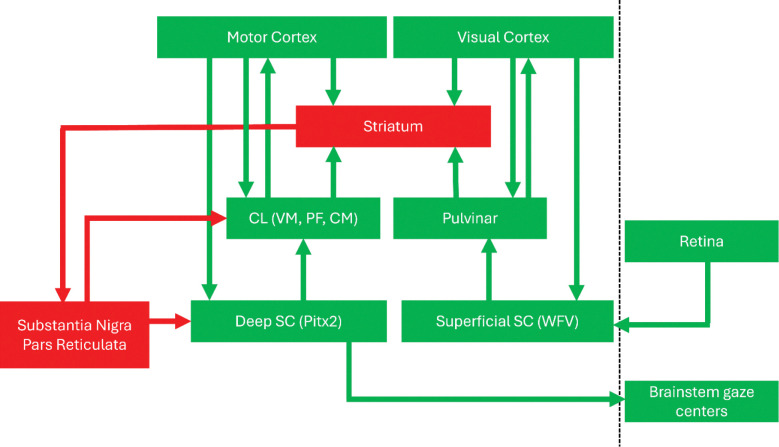
Connections of the tectorecipient thalamus. The superficial layers of the SC receive input from the retina and visual cortex and WFV neurons project to the pulvinar nucleus (which projects to the striatum and is reciprocally connected with visual cortical areas). The superficial SC also projects to the dLGN (not shown). Pitx2 neurons in the deep layers of the SC receive input from the motor cortex and substantia nigra pars reticulata, and project to contralateral brainstem gaze centers as well as ipsilateral thalamic nuclei (CL, VM, PF, CM) that receive input from the substantia nigra pars reticulata, project to the striatum, and are reciprocally connected with the motor cortex. Thus, tectorecipient thalamic nuclei are well positioned to influence movement control. The results of the current study indicate that Pitx2 SC neurons innervate the CL nucleus with large terminals that elicit large amplitude postsynaptic responses and converge with inputs from layer 5 motor cortex inputs to innervate single CL neurons. CL- centrolateral nucleus of the thalamus, VM- ventral medial nucleus of the thalamus, PF- parafascicular nucleus, CM- centromedian nucleus, WFV- widefield vertical, dLGN - dorsal lateral geniculate nucleus. Dotted line indicates the midline.

The thalamic projections of the SC reflect the categorization of the superficial and deep layers as visual and motor respectively (May 2006; Basso et al., 2021); the superficial SC projects to the visual thalamus (dorsal lateral geniculate and pulvinar nuclei; Zhou et al., 2017; Bickford et al; 2015; Whitley et al, 2025) while the deep SC projects to thalamic nuclei considered either motor or “intralaminar” (e.g. the parafascicular, ventromedial, centromedian, and CL nuclei; Krout et al., 2001; Yamasaki et al.,1986). Finally, with the exception of the dorsal lateral geniculate nucleus, all tectorecipient thalamic nuclei project to the striatum (Day-Brown et al., 2010; Zhou et al., 2017; Trevo et al., 2016; Harting et al.,2001),and all thalamic nuclei that receive input from the deep SC layers also receive input from the substantia nigra pars reticulata (SNr, Gerfen et al., 1982). Thus, tectorecipient thalamic nuclei are well positioned to influence sensory-guided movements (Figure 8).

Within this framework, SC projections to the motor/intralaminar thalamus are of particular interest because they can provide copies of motor commands. Such signals are referred to as corollary discharge (CD) or efference copies, which can alert the brain of an impending movement (Fukutomi and Carlson 2020; Crapse and Sommer 2008). One of the best examples of this concept is provided by studies of the primate mediodorsal (MD) nucleus, which receives SC input to its most lateral (paralaminar, Golden et al., 2016) regions and/or the adjacent CL nucleus (Harting et al., 1980; Benevento and Fallon 1975). Neurons that respond to electrical stimulation of the SC are recorded in the lateralmost regions of the primate MD, adjacent to the internal medullary lamina (Wurtz and Sommer 2004) and neurons in these regions display pre-saccadic responses similar to those recorded in the deep layers of the SC (Cavanaugh et al., 2020). In monkeys that were trained to make sequential saccadic eye movements, inhibition of the MD nucleus resulted in deficits in the trajectory of eye movements, consistent with the idea that the thalamus alerts the cortex of impending eye movement so that subsequent movements can be adjusted accordingly (Sommer and Wurtz 2008). To further explore the link between the tectorecipient thalamus and motor control, and relate our findings to these pioneering primate studies, we investigated synaptic circuits within equivalent regions of the mouse thalamus, which we and others define as the CL nucleus (Kuroda and Price 1991; Masullo et al., 2019; Krout et al., 2001; Yamasaki et al., 1986; Graham and Berman 1981).

### Ultrastructure and synaptic properties of SC and L5-MCtx projections to the CL

Neurons in the deep layers of the SC branch to innervate the ipsilateral thalamus and contralateral brainstem and spinal cord (Bickford and Hall 1989; Isa et al., 2020). In the mouse, these neurons are labeled in the Pitx2-Cre line, and their optogenetic activation initiates eye and head movements that vary in amplitude and direction dependent on the location of the light presented in the SC (Masullo et al., 2019). These cells likely correspond to SC neurons that fire with a burst prior to eye movements (Munoz and Wurtz, 1995), which include tectoreticular neurons identified via antidromic stimulation (Rodgers et al., 2006; Guitton and Munoz 1991; Munoz et al., 1991; Munoz and Guitton 1991; Olivier et al., 1993).

We found that projections from Ptix2 premotor neurons form very large synaptic terminals within the CL nucleus. We were particularly interested in comparing these terminals to those that originate from layer 5 neurons in the MCtx since corticothalamic neurons in L5-MCtx exhibit activity related to movement preparation (Economo et al., 2018) and also branch to innervate the SC as well as more caudal motor centers in the brainstem and spinal cord (Kita and Kita 2012; Deschenes et al., 1994). Thus, like SC premotor neurons, L5-MCtx neurons have also been proposed to provide CD or efference copies of motor commands (Guillery and Sherman 2002; Usrey and Sherman 2019). In addition, layer 5 corticothalamic projections have been proposed to drive the activity of thalamic neurons (Sherman and Guillery 1998).

We found that L5-MCtx projections to CL formed terminals that were smaller than Pitx2 terminals and contacted dendrites that were smaller than those contacted by Pitx2 terminals. Thus, on the dendritic arbors of CL neurons, L5-MCtx terminals are likely located distal to Pitx2 inputs. We found that photoactivation of both layer L5-MCtx and Pitx2 inputs elicited responses in CL neurons that depressed in a frequency-dependent manner, indicating that each terminal type exhibits a high probability of neurotransmitter release (Turner and Salt 1998). However, when terminal density was considered, photoactivation of Pitx2 terminals initiated larger amplitude responses in CL neurons when compared to those elicited by photoactivation of L5-MCtx terminals. Thus, SC projections signaling impending head movements provide the most prominent input to CL neurons, as least in terms of the impact of each synaptic terminal.

### Convergence of ascending and descending motor input to single CL neurons

Our dual opsin experiments revealed that individual CL neurons receive potent ascending input from SC premotor neurons as well as descending input from L5-MCtx neurons, both of which may provide CD signals that can drive thalamic activity. Previous *in vitro* studies of the motor thalamus have also revealed convergence of MCtx, basal ganglia, and/or cerebellum inputs (Koster and Sherman 2024; Schafer et al., 2021). In addition, convergence of layer 5 cortical and subcortical inputs has also been detected in somatosensory and visual thalamic nuclei (Groh et al, 2014; Whitley et al., 2025).

In the sensory thalamus, it has been suggested that such convergence serves to report relative timing differences between sensory events and ongoing cortical activity. In the case of the CL nucleus, cortical and subcortical convergence may instead report timing differences between signals related to motor preparation and initiation, with coincident signals potentially boosting movement execution via projections to the MCtx as well as to the striatum (Groh et al., 2008; Deschenes et al., 1996; Wang and Shyu 2004). In fact, tectoreticular cells receive direct input from the MCtx (Zingg et al., 2017, Masullo et al., 2019) and indirect striatal input via the substantia nigra pars reticulata (SNr; Bickford and Hall 1992; Masullo et al., 2019; Villalobos and Basso 2022). Thus, ascending and descending motor signals that converge on individual CL neurons may ultimately fine-tune SC premotor signals. In this way, the tectorecipient thalamus may provide an important feedback loop for ongoing adjustments of movement planning and initiation.

## Supplementary Material

Supplement 1

## Figures and Tables

**Table 1:** Experimental design and statistical analysis

Anatomical analyses (confocal microscopy)						
Mouse line	Injection(s)	Animal numbers	Antibody labeling	Analysis	Figure	Statistical tests
Pitx2-Cre	SC, unilateral	1M, 1F	GFP	Pitx2 terminal distribution	1, S2	
BLK6	AAV in SC and DO-DIO in CL	0M, 3F	GFP, DsRed,	Pitx2-recipient CL cell projections	1	
Pitx2-Ai9	AAV in M1	0M, 3F	GFP, DsRed	L5-MCtx terminal distribution, L5-MCtx terminal distribution in relation to Pitx2 cells in the SC	1, S2	
Anatomical analyses (electron microscopy)						
Mouse line	Injection(s)	Animal numbers	Antibody labeling	Analysis	Figure	Statistical tests/Software
Pitx2-Cre	AAV in SGI, unilateral	2M, 2F	GABA	Size of terminal and postsynaptic dendritic profiles (n=262)	2, S3	Kruskal-Wallis test; ImageJ
C57BL/6J	BDA SC, unilateral		GABA	Size of terminal and postsynaptic dendritic profiles (n=256)	2, S3	Kruskal-Wallis test; ImageJ
Rbp4-Cre	AAV in MCtx, unilateral	3M, 1F	GABA	Size of terminal and postsynaptic dendritic profiles (n=221)	2, S3	Kruskal-Wallis test; ImageJ
*In-vitro* electrophysiology and optogenetics						
Mouse line	Injection(s)	Animal numbers	Antibody labeling	Analysis	Figure	Statistical tests
Pitx2-Ai32	-	2M, 2F, 15 slices	GFP, streptavidin	CL cell optogenetic response and location (n=48) to Pitx2 input simulation; pixel density of the terminal fields	3,5,7, S4, S6	Kruskal-Wallis Test, Mann-Whitney t-test
Pitx2- Ai9	Cre-dependent AAV in SC	3M, 3F, 16 slices	GFP, streptavidin	CL cell optogenetic response and location (n=68) to Pitx2 input simulation: pixel density of the terminal fields	3,5,7, S6	Kruskal-Wallis Test, Mann-Whitney t-test
Rbp4-Cre	Cre-dependent AAV in MCtx	8M, 6F, 25 slices	Streptavidin	CL cell optogenetic response and location of CL cells (n=128) to L5- MCtx, SC, and L5-MCtx +SC pixel density of the terminal fields	4,5,6,7, S4, S5	Kruskal-Wallis Test

**Table 2:** Number of animals and cells for *in-vitro* whole cell patch clamp recordings

Mouse Line	Virus injection to express ChR2	Virus injection to express Chrimson	CL neurons recorded	CL SC Pitx2 responses >100pA	CL SC Pitx2 responses 10-100pA	CL SC only responses >100pA	CL SC only responses 10-100pA	CL L5-MCtx only responses >100pA	CL L5-MCtx only responses 10-100pA	CL responses to SC + L5-MCtx
Pitx2-Ai9	SC; n=6	None	68	19	18					
Pitx2-Ai32	None	None	48	11	7					
Rbp4-Cre	SC: n=3	MCtx: n=3	36			7	5	1	1	3
Rbp4-Cre	MCtx: n=6	SC: n=6	65			1	7	6	11	12
Rbp4-Cre		MCtx: n=2	10					1	3	
Rbp4-Cre										
Rbp4-Cre	SC: n=3		17			5	5			
Rbp4-Cre										
TOTAL cells recorded			244	30	25	13	17	8	15	15

**Table 3:** Viral serotypes, plasmid number, and titer

Virus	Serotype	Plasmid number	Titer (vg/mL)
pAAV-Ef1a-DIO-dApex2	9	117174	3 × 10^13^
pAAV-Ef1α-DIO-hChR2 (H134R) -EYFP-WPRE.hGH.pA	9	20298	≥1x10^13
rAAV. Ef1α.DO_DIO.TdT_EGFP.WPRE.pA	2	37120	5x10^12
pAAV9-Syn-Chrimson-Tdt	9	59171	≥1x10^13
pAAV-hSyn-DIO-EGFP	1	50457	≥7x10^12
pENN.AAV.hSyn1.WPRE.hGH	1	105553	≥7x10^12
AAV1-hSyn-DIO-EGFP	1	50457	≥7x10^12
pAAV-Syn-Flex-rc [ChrimsonR-tdTomato]	5	62723	5x10^12
pAAV-hSyn-hChR2 (H134R)-EYFP	9	26973	≥1x10^13

## Data Availability

The data that support the findings of this study are available upon request from MEB.

## Code availability:

We used available software to analyze the data. No new code was developed for this study.
