# Supplementary material for: Ascending and descending motor pathways converge in the centrolateral nucleus of the thalamus
Source: Res Sq. 2026 Jan 19:rs.3.rs-8264428. Preprint. [Version 1] doi: 10.21203/rs.3.rs-8264428/v1 (PMC12869648; doi:10.21203/rs.3.rs-8264428/v1)
Supplement: Supplement 1 [file NIHPPrs8264428v1-supplement-1.pdf]

Supplemental figures

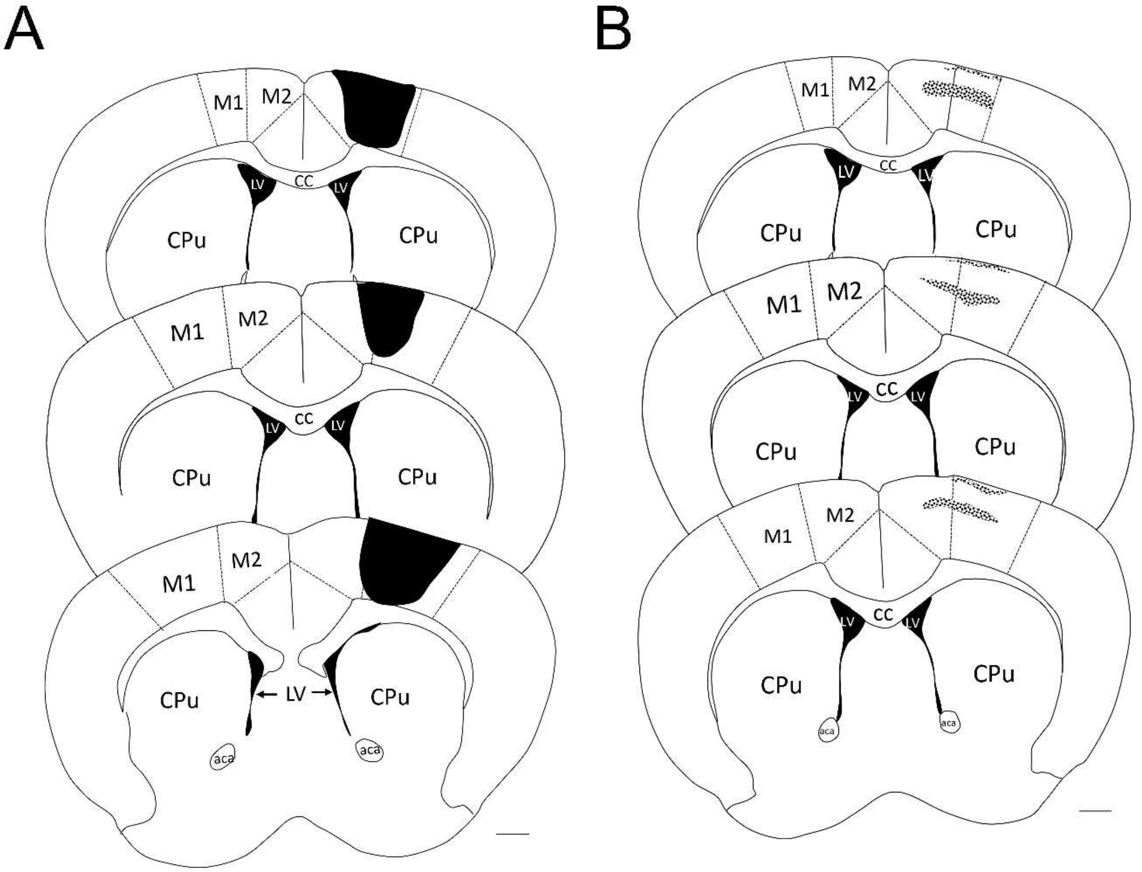

Figure S1: Schematics of injection sites in the motor cortex (A, black, see also Figure 1D inset) and projections from the tectorecipient CL nucleus to the motor cortex (B, black stipple, see also Figure 1I) plotted based on confocal images of experimental tissue. A) Schematic of cortical terminals in L5-MCtx area from tectorecipient CL cells following a TS-Cre virus injection in the SC and a second injection of a Cre-dependent

virus in the CL of C57BL/6J mice shown in Figure 1G-I. B) Schematic of injection site in the L5-MCtx of Pitx2-Ai9 mice that resulted in labeled terminals in the CL, SGI, and Str shown in Figure 1D-F. Scale bars: 100μm.

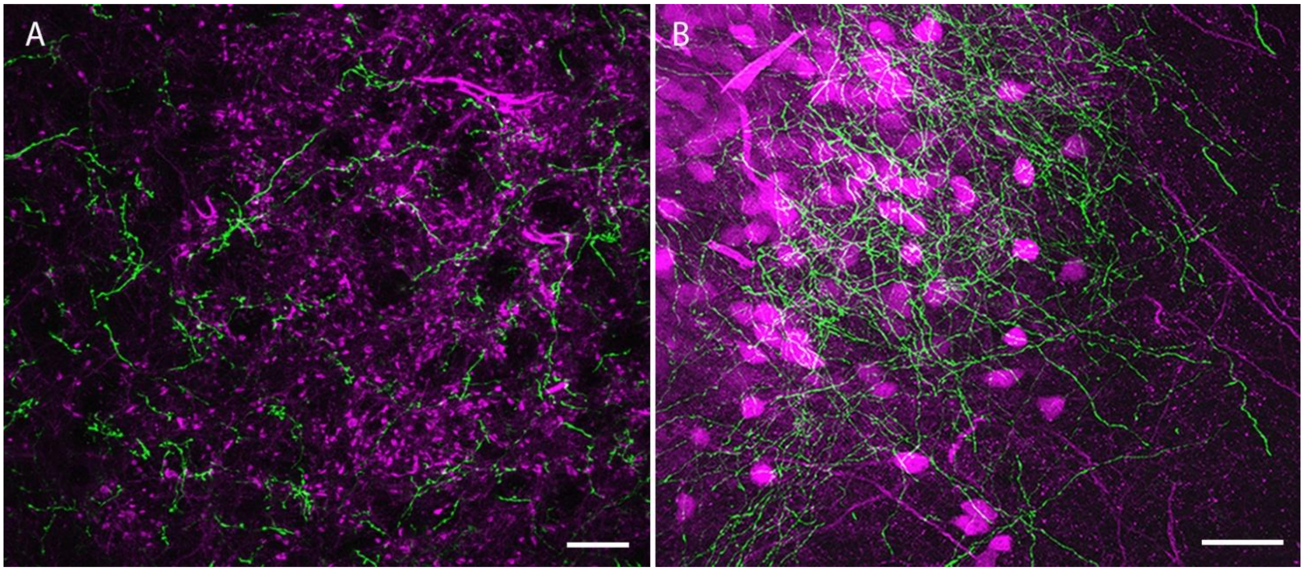

Figure S2: MCtx of a Pitx2-Ai9 mouse was injected with virus to induce the expression of EYFP. A) MCtx (green) and Pitx2 (magenta) terminals in the CL. B) MCtx terminals (green) surrounding Pitx2 cells in the SC. Scale bar in A: 20μm and in B: 50 μm.

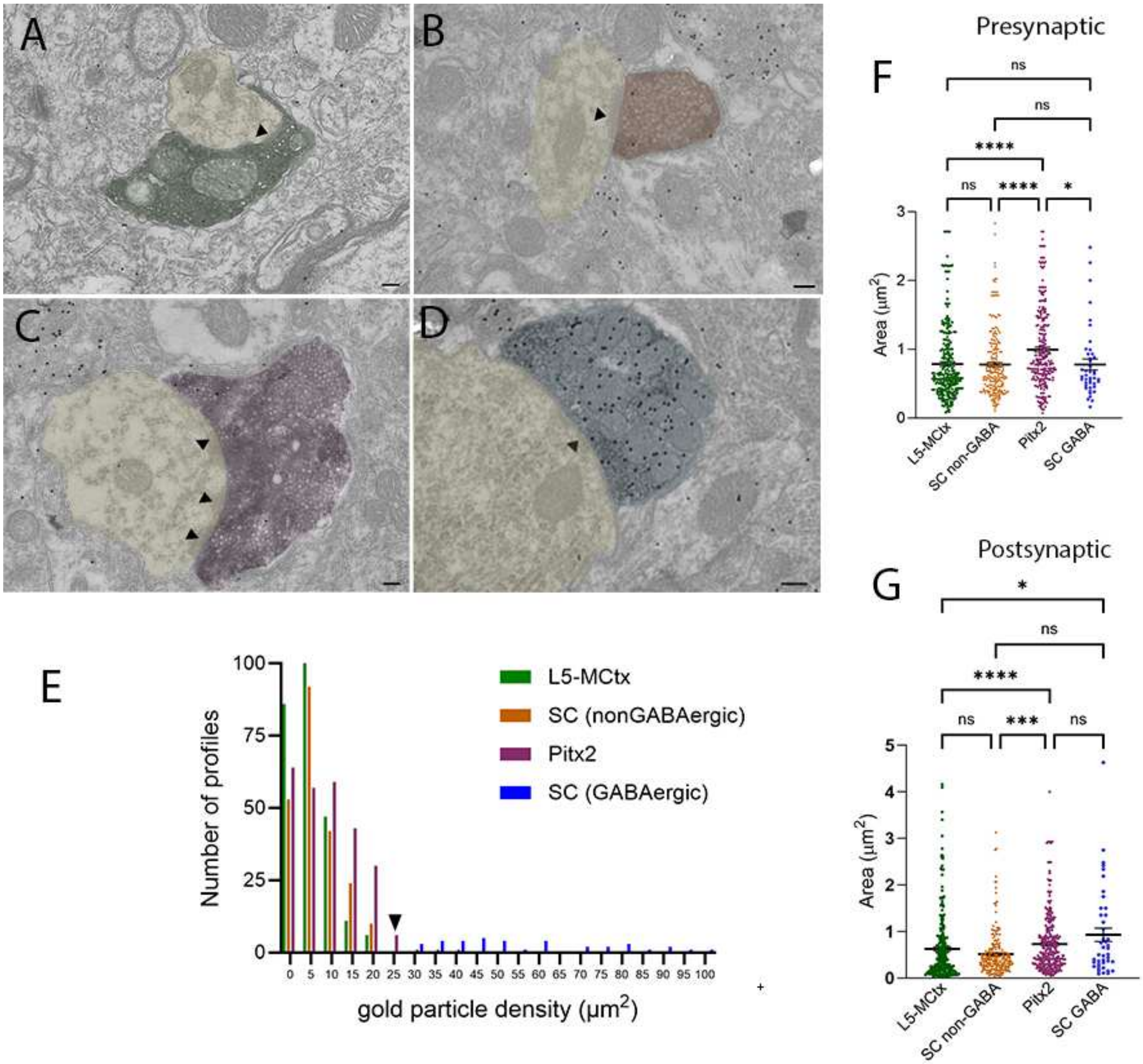

Figure S3: A-D) Example images of labeled L5-MCtx (A, green overlay) nonGABAergic SC (B, orange overlay) Pitx2 (C, purple overlay), and GABAergic SC synaptic terminals (D, blue overlay). Synapses are indicated with black arrowheads and postsynaptic dendrites and indicated with yellow overlays. E) Frequency distribution of the density of gold particles overlying L5-MCtx (green bars), SC (orange and blue bars), and Pitx2 terminals (purple bars). We used the average + 2X the standard deviation of the gold particle density overlying Pitx2 terminals as the cutoff value for determining GABAergic (>24 gold particles/um^2) vs nonGABAergic (<24 gold particles/um^2) profiles in the CL

(indicated by the black arrowhead). F, G) Comparison of the sizes of presynaptic profiles (F) and their postsynaptic targets (G). Presynaptic Pitx2 terminals are significantly larger than L5-MCtx (p<0.0001), nonGABAergic SC (p<0.0001), and GABAergic SC (p=0.0364) terminals in the CL. G) The postsynaptic dendritic partners of Pitx2 terminals were significantly larger than those postsynaptic to L5- MCtx (p<0.0001) and nonGABAergic SC (0.0002) terminals, but not GABAergic SC terminals (p>0.9999). Kruskal-Wallis test was used for statistical analysis and shown are the mean and standard errors of the mean. Scale bars: 300nm (A, B, C, D). Color code in E applies to all panels.

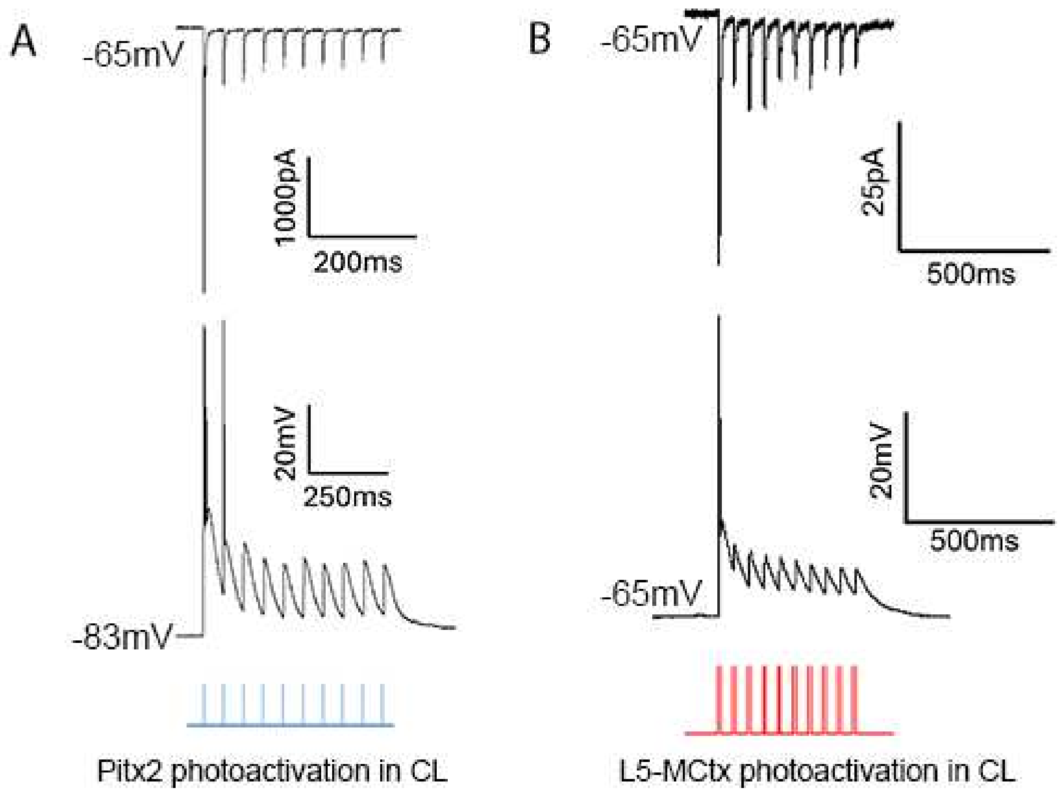

Figure S4: A) Photoactivation of Pitx2 terminals (light pulses shown as blue ticks) occasionally elicited action potentials in response to the first light pulse (n=4) and were

visible in both voltage (top trace) and current (bottom trace) clamp; these were excluded from the analysis of frequency-dependent response and comparison of response amplitude in Figures 3D and 5. B) The photoactivation of L5-MCtx terminals (light pulses shown as red ticks) also occasionally generated action potentials in postsynaptic CL neurons (n=3) that were visible in both voltage (top trace) and current (bottom trace) clamp; these were excluded from the analysis of frequency-dependent response and comparison of response amplitude in Figures 4D and 5.

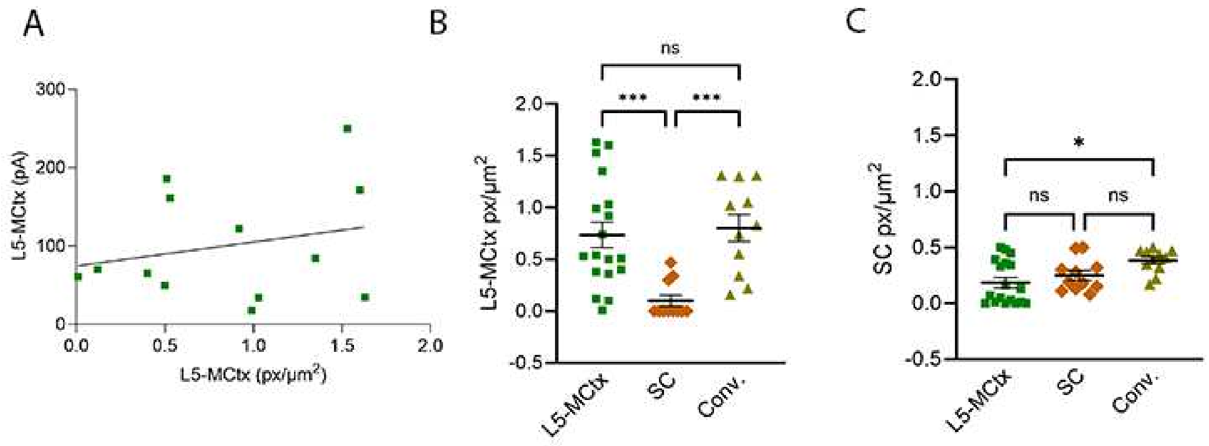

Figure S5 A) Non-linear curve fitting regression analysis of response amplitudes vs pixel densities for CL cells that responded to photoactivation of L5-MCtx terminals. There is a trend for increased EPSC amplitude with increased terminal density around the CL cells. B) Comparison of the densities of L5-MCtx terminals surrounding CL cells that responded to SC only, L5-MCtx only, or both SC and L5-MCtx (conv). Statistical analysis showed a significant difference in pixel densities surrounding cells that responded to L5-MCtx only or SC only ($p=0.0004$) but not between those that responded to L5-MCtx only or L5-MCtx and SC ($p>0.9999$). C) Comparison of the densities of SC terminals surrounding CL cells that responded to SC only, L5-MCtx only, or L5-MCtx+SC (conv). Statistical analysis showed a significant difference in SC pixel

densities surrounding cells that responded to L5-MCtx only or L5-MCtx and SC (p=0.0122) but not between those that responded to SC versus conv. (p=0.2740) or SC versus L5-MCtx (p=0.9645). Kruskal-Wallis test was used in B and C.

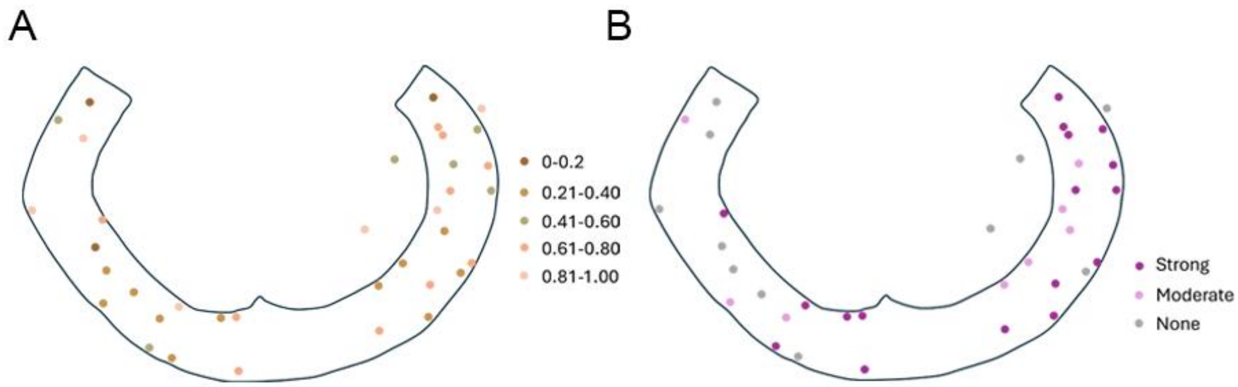

Figure S6: A) Location of CL cells responsive to optogenetic activation of Pitx2 terminals; dots are color coded to indicate their dendritic orientation indices (DO*i*). B) The same cells plotted in A are shown with the color of dots coded to indicate response strength.

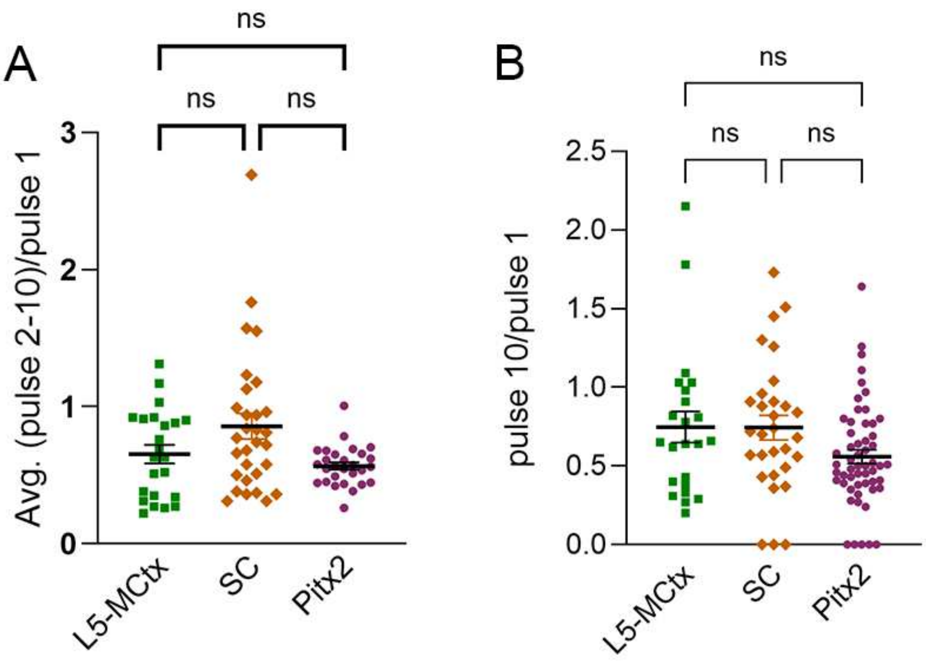

Figure S7: A) The average response amplitudes to pulses 2-10 of a 20Hz train divided by the amplitude of the response to pulse 1 for each CL cell that responded strongly or moderately to the photoactivation of L5-MCtx (n=23), SC (n=30) or Pitx2 (n=55) terminals. We found no significant difference in the frequency-dependence of L5-MCtx vs SC (p=0.4817), L5-MCtx vs Pitx2 (p>0.9999), or SC vs Pitx2 (p=0.1236) responses. B) We found no significant differences in the frequency- dependence of CL cells to pulse 10 divided by the response amplitude to pulse among photoactivated L5-MCtx, SC, or Pitx2 terminals. L5-MCtx vs SC (p>0.9999), L5-MCtx vs Pitx2 (p=0.3749), or SC vs Pitx2 (p=0.0743) responses.
